# Anti-cancer effect of danshen and dihydroisotanshinone I on prostate cancer: targeting the crosstalk between macrophages and cancer cells via inhibition of the STAT3/CCL2 signaling pathway

**DOI:** 10.18632/oncotarget.14958

**Published:** 2017-02-01

**Authors:** Ching-Yuan Wu, Yao-Hsu Yang, Yin-Yin Lin, Feng-Che Kuan, Yu-Shin Lin, Wei-Yu Lin, Ming-Yen Tsai, Jia-Jing Yang, Yu-Ching Cheng, Li-Hsin Shu, Ming-Chu Lu, Yun-Ju Chen, Kuan-Der Lee, Hong-Yo Kang

**Affiliations:** ^1^ Department of Chinese Medicine, Chiayi Chang Gung Memorial Hospital, Chiayi, Taiwan; ^2^ School of Chinese medicine, College of Medicine, Chang Gung University, Tao-Yuan, Taiwan; ^3^ Department of Hematology and oncology, Chiayi Chang Gung Memorial Hospital, Chiayi, Taiwan; ^4^ Department of Pharmacy, Chiayi Chang Gung Memorial Hospital, Chiayi, Taiwan; ^5^ Department of Urology, Chang Gung Memorial Hospital at Chiayi, Puzi City, Taiwan; ^6^ Chang Gung University of Science and Technology, Chia-Yi, Taiwan; ^7^ Department of Chinese Medicine, Chang Gung Memorial Hospital-Kaohsiung Medical Center, Kaohsiung, Taiwan; ^8^ Graduate Institute of Clinical Medical Sciences, Chang Gung University, College of Medicine, Kaohsiung, Taiwan; ^9^ Hormone Research Center, Department of Obstetrics and Gynecology, Kaohsiung Chang Gung Memorial Hospital and Chang Gung University, College of Medicine, Kaohsiung, Taiwan

**Keywords:** dihydroisotanshinone I, STAT3, prostate cancer, Skp2, CCL2

## Abstract

Danshen (Salvia miltiorrhiza Bunge) is widely used in traditional Chinese medicine. In our study, the *in vivo* protective effect of danshen in prostate cancer patients was validated through data from the National Health Insurance Research Database in Taiwan. *In vitro*, we discovered that dihydroisotanshinone I (DT), a bioactive compound present in danshen, can inhibit the migration of both androgen-dependent and androgen-independent prostate cancer cells. In addition, we noted that DT substantially inhibited the migratory ability of prostate cancer cells in both a macrophage-conditioned medium and macrophage/prostate cancer coculture medium. Mechanistically, DT both diminished the ability of prostate cancer cells to recruit macrophages and reduced the secretion of chemokine (C-C motif) ligand 2 (CCL2) from both macrophages and prostate cancer cells in a dose-dependent manner. Moreover, DT inhibited the protein expression of p-STAT3 and decreased the translocation of STAT3 into nuclear chromatin. DT also suppressed the expression of tumor epithelial–mesenchymal transition genes, including RhoA and SNAI1. In conclusion, danshen can prolong the survival rate of prostate cancer patients in Taiwan. Furthermore, DT can inhibit the migration of prostate cancer cells by interrupting the crosstalk between prostate cancer cells and macrophages via the inhibition of the CCL2/STAT3 axis. These results may provide the basis for a new therapeutic approach toward the treatment of prostate cancer progression.

## INTRODUCTION

Prostate cancer is the most common malignant disease and the second leading cause of death among male cancer patients in the United States. The primary treatment for prostate cancer is surgical castration through androgen ablation therapy (ADT) or radiation therapy. However, as many as 50% of prostate cancer patients undergoing radiation therapy experience tumor recurrence and progression within 5 years of treatment completion [1, 2]. These therapies result in castrated levels of testosterone and are temporarily effective in most patients with advanced prostate cancer. Prostate cancer that progresses despite ADT treatment is classified as castration-resistant prostate cancer (CRPC) [3–6]. Several drugs, including sipuleucel-T, abiraterone acetate, and enzalutamide [7], have been approved for treating CRPC, but the effectiveness of these medications is limited, and none of them are curative. Therefore, developing treatment regimens with superior effectiveness and minimal adverse effects for CRPC remain a priority in prostate cancer research.

Tumor-associated macrophages (TAMs) are derived from peripheral blood monocytes that are recruited into the tumor. The tumor-promoting functions of macrophages at the primary site include supporting tumor-associated angiogenesis and promoting tumor cell invasion, migration, and intravasation. Macrophages also potentiate the seeding and establishment of metastatic cells [8]. In a recent study, the number of TAMs was higher in cancer cores than in prostatic intraepithelial neoplasia and benign tissue, and was higher in high-grade prostate cancer, suggesting that TAMs have a role in prostate cancer development [9].

CC chemokine ligand 2 (CCL2), also known as monocyte chemoattractant protein-1, was first identified by its ability to attract monocytes, and it is primarily secreted by monocytes, macrophages, and dendritic cells [10, 11]. CCL2 recruits prostate cancer cells to the bone microenvironment and activates their proliferation rate [12, 13]. Additionally, increased CCL2 expression in prostate cancer cells encourages metastasis through macrophage recruitment [14–17]. S-phase kinase-associated protein 2 (Skp2) belongs to the F-box protein family and is one of the components of the SCF E3 ubiquitin ligase complex. One recent study demonstrated that Skp2 can activate RhoA transcription to stimulate cell migration and invasion [18]. Skp2 is overexpressed in human prostate cancers, which exerts critical downstream effects on STAT3 in human prostate cancer [19, 20]. In addition, CCL2/STAT3 has been shown to stimulate prostate cancer progression [21–23]. Together, emerging evidence suggests that targeting the CCL2/STAT3–Skp2 pathway may offer therapeutic benefits to patients with prostate cancer.

Traditional Chinese medicine (TCM), including acupuncture, traumatology manipulative therapies, and decoction, is a crucial aspect of in health care in Taiwan, as well as other Asian and Western countries. In Taiwan, the National Health Insurance (NHI) program reimburses claims for finished herbal products (FHPs), including single herbs and herbal formulae of TCM. Because the National Health Insurance Research Database (NHIRD) in Taiwan contains the clinical drug and TCM-related information of patients, it is a suitable research database for investigating the efficacy of clinical drugs and TCM [24–26]. Danshen, the dried root of Salvia miltiorrhiza Bunge, has been used to treat numerous cardiovascular and endocrine diseases, including coronary artery disease, angina pectoris, hepatitis, and menstrual disorders [27]. Cryptotanshinone (CT), dihydroisotanshinone I (DT), tanshinone IIA (T2A), and tanshinone I (T1) are four major diterpene compounds of tanshinones in danshen [28]. In addition to their functions in the cardiovascular system, these tanshinones have recently been shown to possess some activity against human cancer cells. Specifically, T1 has inhibited the growth of leukemia and lung and colorectal cancer cells *in vitro* [29–31], T2A has inhibited the growth of breast cancer, glioma, leukemia, and hepatocellular carcinoma cells *in vitro* [32–35], and CT inhibited the growth of hepatocarcinoma cells *in vitro* [36]. Moreover, DT, which contains an abietane-type diterpene quinone (Supplementary Figure 1A), has a protective effect against menadione-induced hepatotoxicity because of its antioxidant properties and its ability to inhibit lipid peroxidation [37]. However, the clinical effects of danshen and the underlying mechanisms of DT on prostate cancer remain unclear.

In this study, we examined the protective effects of danshen and its compounds against prostate cancer. First, to investigate these effects *in vivo*, we analyzed the survival rate of prostate cancer patients by using data obtained from the NHIRD. *In vitro*, we observed the effects of DT on the interaction between macrophages and prostate cancer cells by interrupting the CCL2 pathway. We determined that DT inhibited the protein expression of p-STAT3 and blocked the translocation of STAT3 into the chromatin, as well as suppressed tumor epithelial–mesenchymal transition (EMT) gene expression.

## RESULTS

### Protective effect of danshen in prostate cancer patients from taiwan

**Figure 1:**
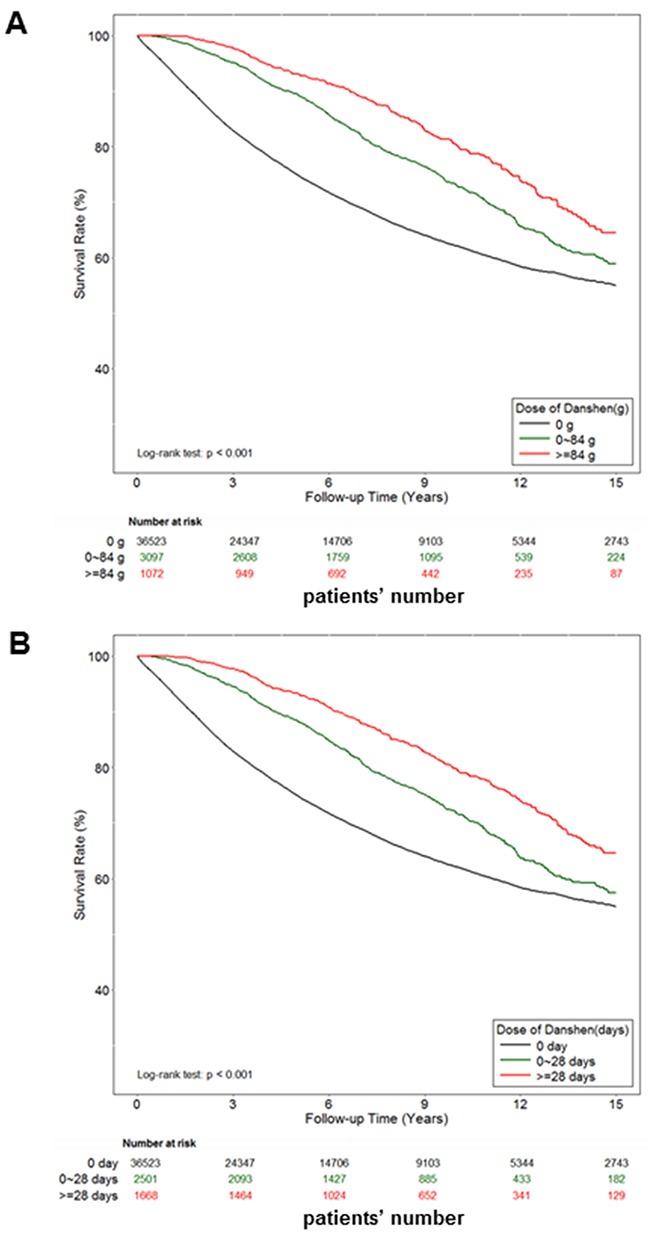
The effect of danshen on the survival rate of Taiwan prostate cancer patients A total of 40,692 prostate cancer patients were included in the study cohort. These patients accrued follow-up time for 15 years. Crude overall Kaplan-Meier survival curves for the prostate cancer patients was investigated. **A**. The patients were categorized into 3 groups: never used danshen, had used danshen more than 84 grams after prostate cancer diagnosed, and those with less than 84 grams danshen used in records. (log-rank: *P*<0.001). **B**. The patients were categorized into 3 groups: never used danshen, had used danshen more than 28 days after prostate cancer diagnosed, and those with less than 28 days with treatment of danshen in records (log-rank: *P*<0.001).

We examined 40,692 patients diagnosed with prostate cancer during the study period. First, patients were categorized into three groups according to drug dosage: those who had never used danshen and those who had used either > 84 or ≤ 84 g of danshen after their prostate cancer diagnosis, as per medical records. Patients were also categorized into three groups according to the duration of their drug use: those who had never used danshen and those who had used danshen for either > 28 or ≤ 28 days after their prostate cancer diagnosis, as per medical records. After 15 years of follow-up, the survival rate analyses demonstrated a strong dose-dependent and time-dependent association between the use of danshen and survival (Figure 1). Notably, danshen users exhibited an increase of 5%–10% in the survival rate compared with danshen nonusers. Thus, these data demonstrated the protective effects for danshen for prostate cancer patients in Taiwan.

### Inhibition of cell motility in various human prostate cancer cells by DT treatment

**Figure 2:**
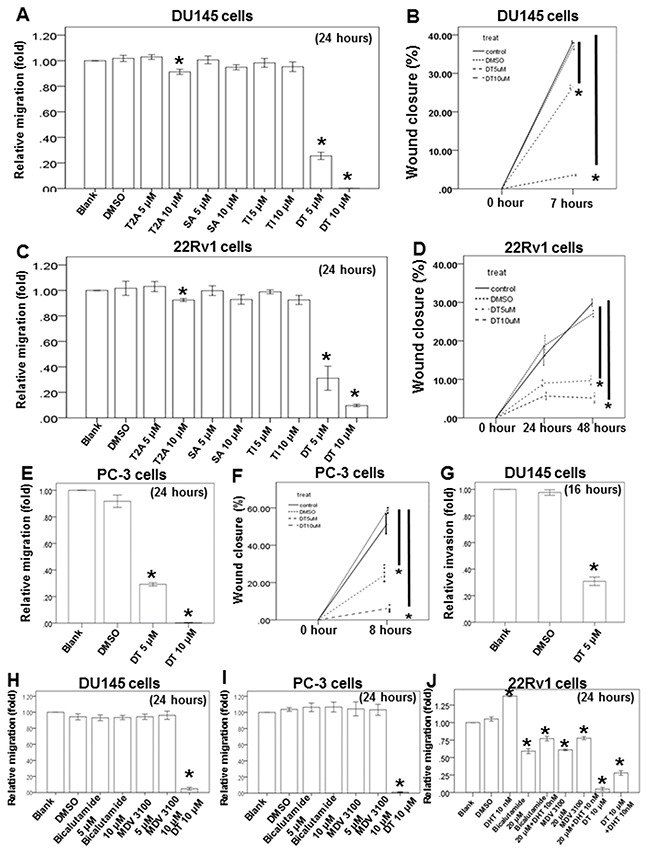
DT block the human prostate cancer cells migration on *in vitro* wound healing assay, transwell migration assay and invasion assay The migration ability of DU145 cells, 22Rv1 cells and PC-3 cells were measured by the transwell migration assay. After treated with indicated drugs for 24 hours, the photographs (× 100) were taken and the migratory cells were measured using AlphaEase®FC StandAlone Software. Numbers of the migratory DU145 cells **A, H**. 22Rv1 cells **C, J**. and PC-3 cells **E, I**. in each group were normalized to the control. The mobility of prostate cancer cells were measured by wound-healing assay. After treatment with indicated drugs, photographs (× 100) were taken. The wound closure of DU145 cells **B**. 22Rv1 cells **D**. and PC-3 cells **F**. were quantified by measuring the remaining unmigrated area using AlphaEase®FC StandAlone Software. The invasion ability of DU145 cells, were measured by the transwell invasion assay. After treated without or with DMSO or DT for 24 hours, the photographs (× 100) were taken and the invasive cells were measured using AlphaEase®FC StandAlone Software. Numbers of the invasive DU145 cells **G**. in each group were normalized to the control. The results were from three independent experiments. (Error bar=mean±S.E.M. Asterisks (*) mark samples significantly different from blank group with *p*<0.05).

In one previous study, more than 50 compounds were isolated from danshen [28]. In general, these compounds can be divided into two groups according to their structure and properties. The first group comprises phenolic acids, such as salvianolic acid B, whose structures contain caffeic acid monomers and oligomers. The second group comprises tanshinones, such as T1, T2A, and DT, whose structures contain abietane diterpenes with a common ortho- or para-naphthoquinone chromophore (Supplementary Figure 1A). To study the effect of these compounds on the migration ability of prostate cancer cells, we examined androgen receptor (AR)-negative (PC-3 and DU145 cells, with a considerably higher invasive capacity) and AR-positive (22Rv1 cells) prostate cancer cells in migration and healing assays and the healing assay (Figure 2). After treatment with the indicated compounds for a specified number of hours, our results revealed that T2A (10 μM) had little inhibitory effect on DU145 cells, and 5–10 μM DT significantly inhibited the migratory ability of three types of prostate cancer cells in a dose-dependent manner (Figure 2A–2F and Supplementary Figure 1B–1G). Moreover, we discovered that 5 μM DT can block the invasion of DU145 cells (Figure 2G).

Next, we used the two current clinically used antiandrogens, bicalutamide and MDV3100, to compare the effects of DT on migration. Our results indicated that 5–10 μM of either bicalutamide or MDV3100 was unable to inhibit the migratory ability of androgen-independent prostate cancer (DU145 and PC-3) cells, whereas 10 μM DT was effectively inhibitory (Figure 2H and 2I). Regarding androgen-dependent prostate cancer cells, the 22Rv1 cells were cultured in RPMI 1640 Medium containing 10% charcoal-dextran-treated fetal bovine serum for at least 72 h before the transwell migratory assay. Our results suggested that 10 nM dihydrotestosterone (DHT) significantly increased the migratory ability of 22Rv1 cells. In addition, 20 μM of either bicalutamide or MDV3100 could inhibit the migratory ability of these cells, even with the cotreatment of 10 nM DHT. Moreover, 10 μM DT had an enhanced inhibitory effect on the migratory ability of 22Rv1 cells, especially with the cotreatment of 10 nM DHT (Figure 2J).

### Effects of DT on prostate cancer cell migration in the macrophage medium and the coculture of prostate cancer cells and macrophages *in vitro*

**Figure 3:**
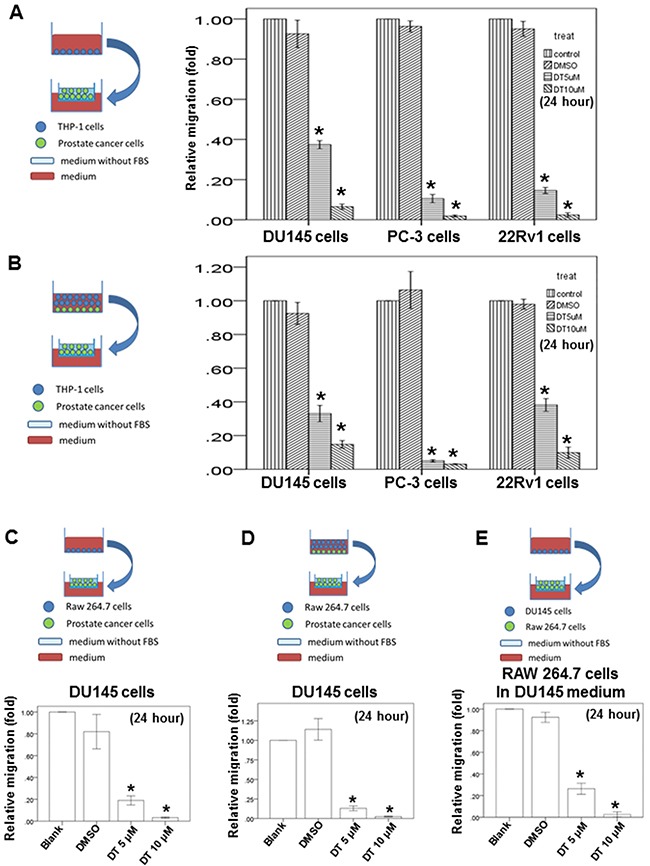
Effects of DT on prostate cancer cells migration in macrophages medium or the prostate cancer/macrophages co-culture *in vitro* model and on RAW 264.7 cells recruitment *in vitro* model The migration ability of human prostate cancers in the macrophages medium or the prostate cancer/macrophages co-culture *in vitro* model were measured by the transwell migration assay. THP-1 cells **A**. or RAW 264.7 cells **C**. were treated with indicated drugs for 24 hours. Then the conditioned medium was collected and placed in the lower chamber. The prostate cancer cells were then placed on the upper chamber for the migration assay. After incubation for 16 hours, the photographs (× 100) were taken and the migratory cells were measured using AlphaEase®FC StandAlone Software. The quantification of the indicated migratory prostate cancer cells numbers in each group were normalized to the control. In the co-culture *in vitro* model, THP-1 cells **B**. or RAW 264.7 cells **D**. and the indicated human prostate cancers were directly mix co-cultured and treated with indicated drugs for 24 hours. Then the conditioned medium were collected and placed in the lower chamber. The indicated prostate cancer cells were then placed on the upper chamber for the migration assay. After incubation for 16 hours, the photographs (× 100) were taken and the migratory cells were measured using AlphaEase®FC StandAlone Software. The quantification of the migratory indicated prostate cancer cells numbers in each group were normalized to the control. For the macrophages’ recruitment ability of human prostate cancer cells, the DU145 cells were treated with indicated drugs for 24 hours. Then the conditioned medium were collected and placed in the lower chamber. The RAW 264.7 cells were then placed on the upper chamber for the migration assay. After incubation for 24 hours, the photographs (× 100) were taken and the migratory cells were measured using AlphaEase®FC StandAlone Software. The quantification of the migratory RAW264.7 cells numbers in each group were normalized to the control **E**. The results were from three independent experiments. (Error bar=mean±S.E.M. Asterisks (*) mark samples significantly different from blank group with *p*<0.05).

Previous studies have demonstrated that macro-phages can promote tumor invasion and metastasis [38], and have revealed that the motility of human prostate cancer cells is inhibited by DT. Therefore, we investigated the effect of DT on the ability of macrophages to promote tumor migration. THP-1 and RAW 264.7 cells have been extensively used to study monocyte–macrophage functions, mechanisms, signaling pathways, and drug activities [39–41]. Herein, after THP-1 cells or RAW 264.7 cells were treated with the control (dimethyl sulfoxide, DMSO) or with either 5 or 10 μM DT for 24 h, the conditioned medium was collected and placed in the lower chambers of the transwell plates (Figure 3A, 3C). Subsequently, the prostate cancer cells were positioned in the upper chambers of the transwell plates with inserts in a serum-free medium for the migration assay. Our results showed that 5–10 μM DT significantly inhibited the migration of prostate cancer cells in a dose-dependent manner in the macrophage medium (Figure 3A, 3C).

Compared with the use of indirect-separate coculture systems in transwell chamber dishes, direct-mixed coculture systems involving macrophages and tumor cells (in which cells communicate with each other through direct contact and which more closely approximate the physiological situation) led to stronger activation signaling by transcription factors, such as STAT3 [42, 43]. Therefore, we examined the ability of prostate cancer cells to migrate under DT treatment in a direct-mixed coculture of prostate cancer and THP-1 cells or RAW 264.7 cells *in vitro* (Figure 3B and 3D). After directly coculturing the macrophages and prostate cancers cells under treatment with DMSO or DT for 24 h, the conditioned medium was collected and placed in the lower chambers of transwell plates (Figure 3B, 3D). Subsequently, the prostate cancer cells were positioned in the upper chambers of the transwell plates with inserts in a serum-free medium for the migration assay. Our results showed that 5–10 μM DT significantly inhibited the ability of prostate cancer cells to migrate in a dose-dependent manner in the direct-mixed coculture medium (Figure 3B, 3D).

### Effects of DT on RAW 264.7 cell recruitment *in vitro*

We also examined the effects of DT on macrophage recruitment by prostate cancer cells. DU145 cells were treated with or without the indicated drugs and with or without the DMSO for 24 h. The conditioned medium was collected and then placed in the lower chambers of the transwell plates. Subsequently, the RAW 264.7 cells were positioned in the upper chambers of the transwell plates in a serum-free medium for the migration assay (Figure 3E). These tests indicated that DU145 cells can activate RAW 264.7 cell migration with or without DMSO treatment. In addition, we found that DT inhibited the migration of RAW 264.7 cells activated by DU145 cells in a dose-dependent manner (Figure 3E).

### Inhibition of CCL2 protein secretion from both prostate cancer cells and macrophages by DT

**Figure 4:**
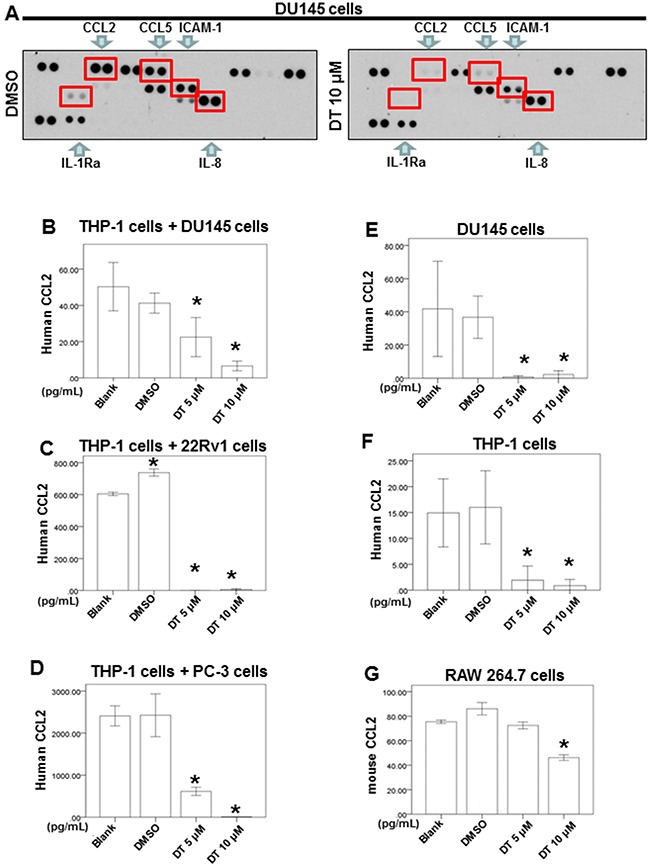
Effects of DT on the proteins secretion from prostate cancer cells and macrophages co-culture *in vitro* For cytokines array, DU 145 cells were treated with DMSO or DT 10 μM for 24 hours. The cultured medium was collected and analyzed by cytokines microarray **A**. Array images were captured following 5-min exposure to X-ray film. For ELISA, the conditioned medium of DU 145 cells **E**. or THP-1 cells **F**. or RAW264.7 cells **G**. or coculture with THP-1 cells/DU 145 cell **B**. or THP-1 cells/22Rv1 cell **C**. or THP-1 cells/PC-3 cells **D**. were collected from untreated cells and cells treated with DMSO or indicated drugs for 24 hours. The secretion of human or mouse CCL2 were measured by ELISA kits (B-G). All the results are representative of at least three independent experiments. (Error bars=mean±S.E.M. Asterisks (*) mark samples significantly different from blank group with *p*<0.05).

Substantial evidence indicates that macrophages promote cancer progression and metastasis. Notably, macrophages are cultured by the tumor microenvironment to stimulate the metastatic process and produce several compounds, including cytokines, that contribute to cancer initiation and promotion [44]. In addition, tumor cells release some molecules, such as chemokines and their receptors, for invasion and migration. Our results indicated that DT has inhibitory effects on the motility of prostate cancer cells and can decrease the ability of prostate cancer cells to recruit macrophages. Moreover, a human cytokine array was performed to identify the differentially expressed cytokines secreted from DU145 cells. After treatment with DMSO or 10 μM DT, we determined that DT treatment attenuated the expression of CCL2, CCL5, interleukin-1 receptor antagonist (IL-1 Ra), and intercellular adhesion molecule 1 (ICAM-1; Figure 4A).

Notably, CCL2 promotes the metastasis of several cancers, including nasopharyngeal carcinoma, bladder cancer, thyroid carcinoma, and colon cancer [45–48]. Additionally, increased CCL2 expression in prostate cancer cells has been demonstrated to encourage metastasis through macrophage recruitment [14–17]. CCL2 also serves as a novel biomarker for prostate cancer [49]. Similarly, one study found that IL-8 is an essential molecule for androgen-independent prostate cancer growth and progression [50]. Thus, we explored the effect of DT on the secretion of IL-8 or CCL2 from prostate cancer cells and macrophages using an enzyme-linked immunosorbent assay (ELISA) to confirm the data from the cytokine array. After treatment with the indicated drugs or DMSO for 24 h, we found that the IL-8 secretion level had not changed in the medium under DT treatment (Supplementary Figure 2A, 2B).

We also discovered that DT treatment inhibited the protein expression of CCL2 in the medium obtained from the prostate cancer cells and macrophage coculture *in vitro* system (Figure 4B–4D). Moreover, we determined that DT inhibited the secretion of CCL2 from the cultured medium of DU145 cells (Figure 4E), THP-1 cells (Figure 4F), and RAW 264.7 cells (Figure 4G).

### Expression of the whole genomic mRNA profile under DT treatment

**Figure 5:**
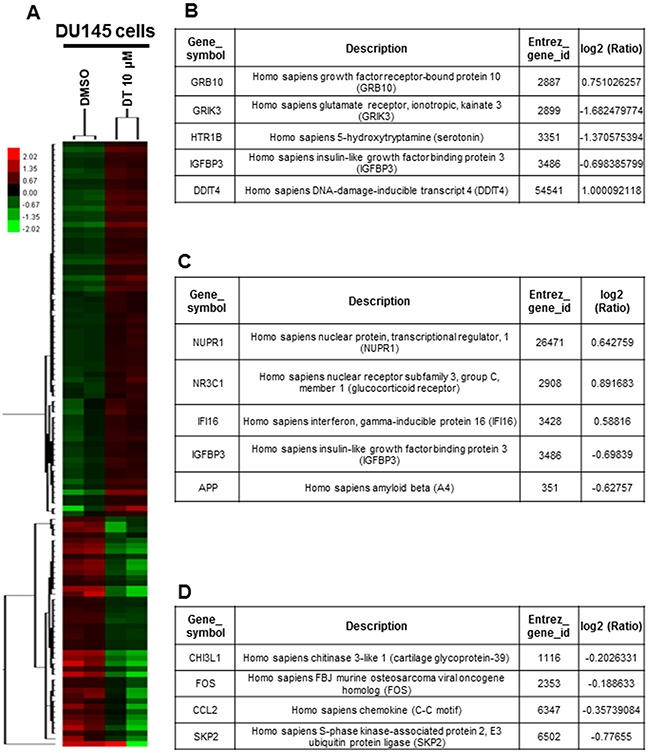
Effects of DT on the whole genomic mRNA expression of DU 145 cells **A**. Hierarchical clustering analysis demonstrated the mRNA expression patterns of DU 145 cells under the treatment of DMSO or DT 10 μM for 24 hours. After gene ontology (GO) enrichment analysis based on biological process, the genes related negative regulation of cell communication (GO:0010648) **B**. and positive regulation of apoptosis (GO:0043065) **C**. **D**. The RNA expression of *CHI3L1, FOS, CCL2* and *skp2* were showed through mRNA array. (Log2 ratio/fold change: the differential expressed level between the DU 145 cells treated with DMSO or DT.)

To evaluate the pathway maps and molecular and cellular functions of the genes correlated with DT in prostate cancer cells, we analyzed the whole genomic mRNA expression profile of DU145 cells treated with DMSO or 10 μM DT for 24 h through an mRNA array (Figure 5A). Following gene ontology (GO) enrichment analysis based on biological processes, 24 pathways were identified (Supplementary Table 1, *p* < 0.005). These pathways included the negative regulation of cell communication (GO: 0010648; Q-value: 6.47169E-01) (Figure 5B), positive regulation of apoptosis (GO: 0043065; Q-value: 7.39806E-01) (Figure 5C), cellular amino acid biosynthetic process (GO: 0008652; Q-value: 1.80621E-03), amine biosynthetic process (GO: 0009309; Q-value: 8.88332E-03), neutral amino acid transport (GO: 0015804; Q-value: 3.02479E-01), serine family amino acid metabolic process (GO: 0009069; Q-value: 3.4819E-01), and amino acid transport (GO: 0006865; Q-value: 3.81235E-01) (Supplementary Table 1). Thus, our data indicate that the mechanism of DT treatment in prostate cancer may involve cell–cell communication, apoptosis, amino acid biosynthesis, and amino acid transport.

**Figure 6:**
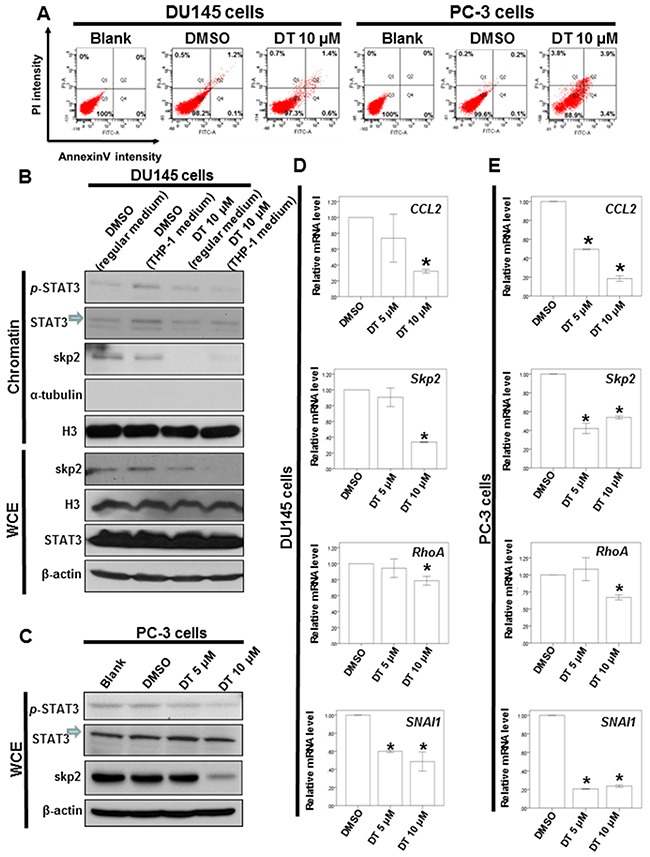
DT induces partial apoptosis and inhibits the protein expression of p-STAT3 and Skp2 protein expression through blocking the migration of STAT3 into chromatin fraction in prostate cancer cells **A**. DU 145 cells or PC-3 cells were treated without or with 10 μM of DT for 24h. Cell apoptosis was detected by flow cytometry with annexin-V-FITC/PI dual staining. The representative histograms of flow cytometric analysis using double staining with annexin-V-FITC (FITC-A) and PI (PI-A). Q1 (annexin-V−/PI+) show necrosis cells; Q2 (annexin-V+/PI+) show the late apoptosis cells; Q3 (annexin-V−/PI−) show normal cells; Q4 (annexin-V+/PI−) show the early apoptosis cells. **B**. DU 145 cells were treated with or without DT or THP-1 conditioned medium. After incubation for 24 hours, the chromatin fraction and total cell extracts were collected for IB analysis. **C**. Total cell extracts of PC-3 cell were harvested from untreated cells and cells treated with DT for 24 hours. The protein was immunoblotted with polyclonal antibodies specific for *p*-STAT3, STAT3 or skp2. β-actin or Histone3(H3) or α-tubulin was used as an internal loading control. Total mRNA was extracted from the DU145 cells **D**. or PC-3 cells **E**. after treat with indicated drugs for 24 hours. The coding regions of human *skp2, RhoA* and *SNAI1* were used as probes for real time polymerase chain reaction analysis. All the results are representative of at least three independent experiments. (Error bars=mean±S.E.M. Asterisks (*) mark samples significantly different from DMSO group with *p*<0.05).

To further analyze the effect of DT on the apoptosis of prostate cancer cells, the DU145 and PC-3 cells were treated with DT or DMSO for 24 h and analyzed for apoptosis through flow cytometry with annexin V/PI dual staining. As illustrated in Figure 6A, 10 μM DT treatments only induce the partial apoptosis of DU145 and PC-3 cells from 0% to 2% and from 0% to 7.3%, respectively, in 24 h. These results suggest that apoptosis may not be the major pathway in prostate cancer cells under DT treatment.

### The expression profiles of the STAT3 signaling pathway were inhibited in prostate cancer cells with DT treatment

Skp2 is a transcriptional target of STAT3 [43], which, along with downstream genes (including RhoA), can stimulate prostate cancer metastasis and proliferation [16, 18, 44, 45]. Previous studies have reported that a combination of STAT3, CCL2, and EMT activates prostate cancer progression and may provide a novel therapeutic target for the prevention of prostate cancer metastasis [21–23]. Moreover, STAT3 is a valuable biomarker for prognosis prediction and a therapeutic target in human solid tumors, including prostate cancer [51]. Although cytokine receptors are activated through cytokines at the molecular level, the signaling effectors and kinases can phosphorylate STAT3 at Tyr705, which results in the translocation of activated p-STAT3 to the nucleus. Subsequently, p-STAT3 can induce the downstream target genes, such as CCL2, CCL5, ICAM-1, SNAI1, chitinase-3-like 1 (CHI3L1), FBJ murine osteosarcoma viral oncogene homolog (FOS), and Skp2, that promote various cellular processes for cancer progression [52–54].

Our results demonstrated that DT could reduce the secretion of chemokines, including CCL2, CCL5, and ICAM-1, in prostate cancer cells, and inhibit prostate cancer cell migration under the macrophages’ cultured medium (Figure 3A). The mRNA array data also indicated that DT treatment can inhibit the mRNA expression of CHI3L1, FOS, Skp2, and CCL2, the downstream genes of p-STAT3 (Figure 5D). Next, we investigated the effect of DT on the protein expression of STAT3. Because the conditioned medium of THP-1 cells can activate the migration of prostate cancer cells (Figure 3A) and contain CCL2 (Figure 4F), we used this medium as our model. After the THP-1 cells were treated with DMSO or 10 μM DT for 24 h, the conditioned medium was collected. Subsequently, the medium of DU145 cells was replaced with the conditioned medium or regular medium and then cultured for 24 h. As depicted in Figure 6B, compared with the regular medium, the conditioned medium of the THP-1 cells induced p-STAT3 protein expression and activated the translocation of p-STAT3 into the chromatin fraction in DU145 cells. Moreover, compared with the conditioned medium treated with 10 μM DT, the translocation of STAT3 and the protein expression of p-STAT3 and Skp2 were inhibited. However, the protein expression of STAT3 was not changed in the whole cell extract under DT treatment (Figure 6B). In PC-3 cells, the protein expression of p-STAT3 and Skp2 in the whole cell extract were also inhibited by DT in a dose-dependent manner (Figure 6C). To confirm the Western blot results, we also found the protein expression of Skp2 was decreased through an immunofluorescence assay in DU145 and PC-3 cells treated with DMSO or 10 μM DT (Supplementary Figure 3A, 3B).

We also used a quantitative polymerase chain reaction (q-PCR) to validate the mRNA expression of the downstream genes. As illustrated in Figure 6D and E, our results revealed that DT inhibited the mRNA expression of CCL2, Skp2, RhoA, and SNAI1 in DU145 cells (Figure 6D) and PC-3 cells (Figure 6E).

## DISCUSSION

To the best of our knowledge, this is the first matched-cohort population study that analyzed the clinical effects of danshen on the survival rate of prostate cancer patients. Specifically, we examined data from the computerized insurance reimbursement claims database in Taiwan, and we used the NHIRD to discover that danshen can prolong the survival rate of prostate cancer patients in Taiwan. However, there were some limitations with this study that should be noted. First, the NHI program offered reimbursements only for FHPs that were prescribed by TCM physicians, and not for decoctions provided by pharmacies; this may have led to an underestimation of the TCM utilization dosage. However, this underestimation could be small because most FHPs were reimbursed and easily obtained from clinical TCM doctors in Taiwan. Second, we could not confirm the exact dosage ingested by this study's patients. We presumed that all medications were taken by patients as prescribed; however, this may have led to overestimation of the actual ingested dosage because some degree of noncompliance is always expected. Although we determined that the use of danshen could prolong the survival rate of prostate cancer patients in Taiwan, more rigorous, randomized, double-blind, and placebo-controlled trials are necessary to confirm the protective effect of danshen in prostate cancer patients in the future.

Abietane diterpenes that have a common ortho- or para-naphthoquinone chromophore are the major components of tanshinones, and include T1, T2A, CT, and DT [28]. In our study, we found that 5 and 10 μM DT inhibited transwell migration and wound-healing migration of DU145, PC-3, and 22Rv1 cells (Figure 2). Furthermore, the wound-healing migration of DU145 and PC-3 cells without any treatment was not robust like the transwell migration. The duration of the wound-healing and transwell assays was approximately 7–8 h and 24 h for DU145 cells and PC-3 cells, respectively. In addition, the cell migration and wound-healing affinity were analyzed in metastatic (DU145 and PC-3 cells) and castration-resistant (22Rv1 cells) prostate cancer cell lines. DU145 and PC-3 cells had a considerably higher invasive capacity than did 22Rv1 cells. After 7–8 h of incubation, the wound closure percentage could reach 40% and 60% in DU145 and PC-3 cells, respectively (Figure 2B, 2F). In 22Rv1 cells, the percentage of wound closure reached 30% in a 48-h treatment (Figure 2D).

In one previous study, T1 was the most potent tanshinone for apoptosis induction in PC-3 cells, increasing apoptosis by 30% at a concentration of 5 μM [55]. By contrast, CT and T2A (at a concentration of 10 μM) only induced apoptosis in approximately 10% of PC-3 cells. Additionally, T2A and DT possess an orthoquinone and an intact ring D. In the present study, we found that 10 μM DT treatments can induce the partial apoptosis of 7.3% of PC-3 cells in 24 h, which is consistent with previous results [55]. Therefore, we suggest that apoptosis may not appreciably effect prostate cancer cells under tanshinones treatment in 24 hours.

Considerable evidence also indicates that macrophages are cultured by the tumor microenvironment to promote metastasis through the production of several compounds, including cytokines [44]. In the present study, we investigated the direct effect of DT on the migratory ability of prostate cancer (Figure 2). We also examined the effect of cytokines from the conditioned medium of THP-1 cells, treated with DMSO or DT, on the migratory ability of prostate cancer cells (Figure 3A). Notably, no cytokines from the THP-1 cells were observed in the medium depicted in Figure 2.

Previously, the importance of direct contact was the focus of investigations regarding cell–cell interactions; for example, STAT3 activation in several types of cancer cells has been identified to be significantly induced by the direct coculture of macrophages and cancer cells [42, 56, 57]. To simulate the physiological interactions between macrophages and tumor cells herein, we used a direct-mixed cell–cell coculture system to investigate the mobility of prostate cancer cells and the signaling pathway (Figure 3B). We directly cocultured THP-1 cells and prostate cancer cells treated with DMSO or DT for 24 h, and then collected the conditioned medium to investigate the effect of cytokines from both THP-1 cells and prostate cancer cells on the migratory ability of prostate cancer cells. Overall, the cytokines from both THP-1 cells and prostate cancer cells were close to the real physiological microenvironment (Figures 2, 3A, and 3B).

RAW 264.7 cells are mouse leukemic monocyte–macrophage cells that are frequently used to study paracrine communication between cancer cells and macrophages [58–60]. Because THP-1 cells are from Homo sapiens, we also used RAW 264.7 cell lines from Mus musculus to repeat our experiments and confirm the effect of DT on macrophages. Specifically, we investigated the effect of cytokines from the conditioned medium of RAW 264.7 cells treated with DMSO or DT on the migratory ability of DU145 cells (Figure 3C). We directly cocultured RAW 264.7 and DU145 cells treated with DMSO or DT for 24 h, and then collected the conditioned medium to investigate the effect of cytokines from both RAW 264.7 and DU145 cells on the migratory ability of prostate cancer. Overall, cytokines from both RAW 264.7 and DU145 cells were close to the real physiological microenvironment (Figure 3C and 3D). Finally, we examined the effect of cytokines from the conditioned medium of DU145 cells treated with DMSO or DT on the migratory ability of RAW 264.7 cells (Figure 3E). In summary, this was a novel study that demonstrates how DT can inhibit the migrating ability of the prostate cancer cells, as well as the macrophage recruitment ability of the prostate cancer cells.

As noted in a previous study, T2A exerts cardioprotective effects through the reduction of CCL2 and TGF-β1 secretion by cardiac fibroblasts [61]; however, the effect of DT on cytokine secretion from prostate cancer cells and macrophages is unclear. Therefore, the human cytokine array for the conditioned media (secretome analysis) of DU145 cells was performed in the present study to identify the differentially expressed cytokines. In our results, DT treatment attenuated the expression of some cytokines, including CCL2, CCL5, IL-1 Ra, and ICAM-1 (Figure 4A). Moreover, through ELISA, we observed that DT inhibited the secretion of CCL2 from the cultured medium of DU145, THP-1, and RAW 264.7 cells.

Previous studies have also indicated that increased CCL2 expression in prostate cancer cells encouraged metastasis through macrophage recruitment [14–17, 49]. From the results of an mRNA array, we similarly observed that the mRNA expression of CCL2 was inhibited in DT-treated DU145 cells (Figure 5B). This suggests that DT may inhibit the migration of both macrophages and prostate cancer cells by blocking several cytokines, including CCL2, which is the principal cytokine during DT treatment.

Scholars have argued that the CCL2–p-STAT3 pathway stimulates prostate cancer metastasis and progression, and may be critical in future efforts to develop new therapeutic approaches for treating prostate cancer [21, 22]. Additionally, EMT-inducing transcription factors, whose activity is activated by STAT3, promote the metastasis of cancer cells to distant organ sites [62–65]. Other prior research has demonstrated that STAT3 interacts with the Skp2 pathway to activate the motility and invasion of cancer cells [66, 67]. Moreover, RhoA GTPase is crucial in cancer metastasis, and Skp2 induces RhoA transcription and consequently promotes cell migration, invasion, and cancer metastasis [18].

According to the cytokine array data in the present study, the expression of CCL2, CCL5, and ICAM-1, which were all downstream target genes of p-STAT3 at Tyr705, decreased. Subsequently, we analyzed the gene expression levels in DT-treated DU145 cells through the whole genomic mRNA array. We also observed the mRNA expression of several STAT3 target genes [54], including CHI3L1 [log2 (Ratio) = −0.2026] and FOS [log2 (Ratio) = −0.1886], which had also decreased (Figure 5D). Furthermore, the mRNAs of Skp2, RhoA, and SNAI1 were identified through q-PCR to be decreased in the DT-treated prostate cancer cells (Figure 6D, 6E). These data suggest that the mRNA expression of the downstream target genes of STAT3 are inhibited by DT.

Notably, STAT3 can be phosphorylated at Tyr705 by activated cytokine receptors and the signaling effectors and kinases, which can result in the translocation of activated p-STAT3 to the nucleus and thereby induce the downstream target genes that promote various cellular processes for cancer progression [52–54]. In a previous study, CT inhibited STAT3 Tyr705 phosphorylation in DU145 prostate cancer cells by binding to the SH2 domain of STAT3 and blocking the formation of STAT3 dimers [68]. In our results, the cultured medium of the THP-1 cells stimulated the migration of prostate cancer cells (Figure 2A), activated the migration of STAT3 into the chromatin fraction, and induced p-STAT3 protein expression in DU145 cells (Figure 6B). However, the migratory ability of STAT3 and protein expression of p-STAT3 and Skp2 were inhibited following DT treatment (Figure 6B). Moreover, the mRNA expression of Skp2 and these downstream EMT genes, including RhoA and SNAI1, were also decreased under DT treatment (Figure 6D, 6E). This suggests that DT may block the activation and migration of STAT3 into the chromatin fraction, and then inhibit the mRNA expression of these downstream genes.

Our mRNA array data also identified 24 pathways in DT-treated DU145 cells through GO enrichment analysis based on biological processes (Supplementary Table 1, *p* < 0.005). Thus, our data suggest that DT treatment in prostate cancer may involve cell–cell communication, apoptosis, amino acid biosynthesis, and amino acid transport. These findings are consistent with previous results regarding apoptosis [55] and the inactivation of STAT3 [68], and extends our understanding of the underlying mechanisms of DT treatment.

In summary, this is a novel study that demonstrated how DT can inhibit the migratory ability of the prostate cancer cells and the macrophage recruitment ability of the prostate cancer cells. These results suggest that DT is a novel anticancer agent in the armamentarium of prostate cancer management.

## MATERIALS AND METHODS

### Data source

We conducted a nationwide cohort study by using population-based data from the Taiwan National Health Insurance Research Database (NHIRD). Because National Health Insurance (NHI) is a compulsory universal program for all residents in Taiwan, the NHIRD is a comprehensive health care database that covers nearly the entire 23.7 million populations of this country. We used databases for admissions and outpatient visits, both of which included information on patient characteristics such as sex, date of birth, date of admission, date of discharge, dates of visits, and up to five discharge diagnoses or three outpatient visit diagnoses (according to International Classification of Diseases, Ninth Revision (ICD-9) codes). The data files also contained information on patient prescriptions, including the names of prescribed drugs, dosage, duration, and total expenditure. Following strict confidentiality guidelines in accordance with personal electronic data protection regulations, the National Health Research Institutes of Taiwan maintains an anonymous database of NHI reimbursement data that is suitable for research. Meanwhile, this study was approved by the Ethics Review Board of Chang Gung Memorial Hospital, Chia-Yi Branch, Taiwan.

### Study subjects

This study cohort was obtained from the Taiwanese National Health Insurance (NHI) research database, which included all patients who received diagnosis of malignant neoplasm of prostate (ICD-9-CM codes:185) in catastrophic illness database between January 1, 1997, and December 31, 2012. Patients who apply for a cancer catastrophic illness certificate are required to provide pathological reports or other supporting documents, such as laboratory and image studies. The date of the initial prostate cancer diagnosis was defined as the index date of prostate cancer. Patient with other cancer diagnosed before prostate cancer or missing data were excluded. A total of 40,692 patients were included in the study cohort. These patients accrued follow-up time beginning on January 1, 1997 and ended on the date of death, or withdrawal from the registry or on December 31, 2013.

### Danshen exposure and potential confounders and statistical analysis for NHIRD

Finished herbal products (FHP) are the modern form of Chinese herbal remedies, of which single herb and herbal formulae are concentrated into granulated compounds, which fully reimbursed under the current NHI system of Taiwan. The list of reimbursed FHP was downloaded from the website of the Bureau of NHI. The corresponding drug information for each FHP including the proportions of each constituent, date and period of approval as drug, code and name of manufacturer. By using this information, we determined the original amounts of danshen, in grams, for each mixture of FHPs. First, patients were categorized into 3 groups: never used danshen (the patients’ number were 36523), had used danshen more than 84 grams after prostate cancer diagnosed (the patients’ number were 1072), and those with less than 84 grams danshen used in records (the patients’ number were 3097). Moreover, patients also were categorized into 3 groups: never used danshen (the patients’ number were 36523), had used danshen more than 28 days after prostate cancer diagnosed (the patients’ number were 1668), and those with less than 28 days with treatment of danshen in records (the patients’ number were 2501). We used the Kaplan-Meier method to estimate survival probabilities and the log-rank test was performed to examine differences in the risk of death in the cohort. All of these analyses were conducted using SAS statistical software (Version 9.4; SAS Institute, Cary, NC, USA).

### Cell migration assay

Cell migration assays were performed as described previously [22]. For the migration of monoculture of human prostate cancer cells, the human prostate cancer cell lines (DU145 cells, PC-3 cells, or 22Rv1 cells) (1 × 10^5^ cells/well) were plated in the upper chambers of Transwell plates with 8-μm pore polycarbonate membrane inserts in a medium without FBS. A medium with FBS was plated in the lower chambers. After treatment with or without DMSO and with indicated concentrations of DT for 16–24 hours, the cells that have migrated into the bottom were fixed and stained using 1% toluidine blue, and the numbers of migratory cells were averaged after counting 6 randomly selected fields. For the prostate cancer recruitment assay, RAW264.7 cells or THP-1 cells (1 × 10^5^ cells/well) were treated with or without DMSO and with an indicated concentration of DT for 24 hours. The conditioned medium or control medium was then collected and plated in the lower chambers. The indicated parental human prostate cancer cells (1 × 10^5^ cells/well) were plated in the upper chambers in the medium without FBS. After incubation for 24 hours, the cells that have migrated into the bottom were fixed and stained using 1% toluidine blue, and the numbers of migratory cells were averaged after counting 6 randomly selected fields. For a prostate cancer cell and macrophage direct mixed co-culture system, the indicated prostate cancer cell line (DU145 cells, PC-3 cells, or 22Rv1 cells) (1 × 10^5^ cells/dish) was plated in a 6-mm dish overnight. After becoming adherent, the indicated macrophages (RAW264.7 cells or THP-1 cells) (1 × 10^5^ cells/dish) were plated in the same dish. After becoming adherent, the direct mixed co-culture system was treated with or without DMSO and with indicated concentrations of DT for 24 hours. Either the conditioned medium or the control medium was then plated in the lower chambers. The indicated parental human prostate cancer cells (1 × 10^5^ cells/well) were plated in the upper chambers in the medium without FBS. After incubation for 24 hours, the cells that have migrated into the bottom were fixed and stained using 1% toluidine blue, and the numbers of migratory cells were averaged after counting 6 randomly selected fields. Each sample was assayed in triplicate, and each experiment was repeated at least twice.

### Wound-healing assay

Wound-healing assays were performed as described previously [69]. An IBIDI culture insert (IBIDI GmbH) consists of two reservoirs separated by a 500 μm thick wall. For the prostate cancer cells migration assay, an IBIDI culture insert was placed into one well of the 12 well plate and slightly pressed on the top to ensure tight adhesion. An equal number of indicated human prostate cancer cells were added into the two reservoirs of the same insert and incubated at 37°C/5% CO_2_ overnight. The insert was then gently removed creating a gap of 500 μm. After treated with or without DMSO or indicated concentration of DT for the indicated duration, the migration of human prostate cancer cells was observed using Nikon TE3000 microscope. Photographs of the same area of the wound were taken after the indicated duration to measure the width of the wound. The wound closure was quantified at indicated hours post-wound by measuring the remaining unmigrated area using AlphaEase®FC StandAlone Software. Each experiment was repeated at least twice.

### Macrophage recruitment assay

Macrophage recruitment analyses were performed as described previously [22]. DU145 cells were treated with DT for 24 hours. The conditioned medium or control medium were collected and plated into the lower chamber of transwell plates with a 5 μm pore polycarbonate membrane insert. RAW264.7 (1 × 10^4^) cells were plated onto the upper chamber for macrophage migration assay. After incubation for 16 hours, the cells that have migrated into the bottom were fixed, stained using 1% toluidine blue, and the numbers of invasive cells were averaged after counting 6 randomly selected fields. Each sample was assayed in triplicate. Each experiment was repeated at least twice.

### Enzyme-linked immunosorbent assay (ELISA)

The ELISA were performed as described previously [36]. Medium was collected from monoculture of prostate cancer cells or macrophages, or from co-cultures of prostate cancer cells and macrophages under the treatment with or without DMSO or indicated concentration of DT for 24 hours. Human or mouse CCL2 or human IL8 in medium were detected by human or mouse CCL2 ELISA kits (eBioscience, catalog number: 88-7399, 88-7391) or human IL8 ELISA kit (eBioscience, catalog number: 88-8086) according to the manufacturer's instructions.

### Chromatin fractionation

Chromatin fractionation was performed as described [70]. In brief, THP-1 cells (1 × 10^5^ cells/well) were treated with the vehicle (DMSO) or with 10 μM DT for 24 hours, the conditioned medium was collected. Then, the medium of DU 145 cells was replaced with the conditioned medium or regular medium and subsequently cultured for 24 hours. After the DU145 cells were treated with indicated conditioned medium for 24 hours, the cells were washed twice by cold-PBS. Cell pellets were resuspended in buffer A (50 mM Hepes, pH 7.9, 10 mM potassium chloride (KCl), 1.5 mM MgCl_2_, 0.34M Sucrose, 10% Glycerol (v/v), 1 mM dithiothreitol (DTT), protease inhibitor cocktail (Roche), 0.1% Triton X-100 (v/v) and phosphatase inhibitor cocktail I and II (Sigma) on ice. After centrifuge, pellets were lysed by buffer B (3 mM EDTA, 0.2 mM ethylene glycol tetraacetic acid (EGTA), 1 mM DTT, protease inhibitor cocktail (Roche) and phosphatase inhibitor cocktail I and II (Sigma) After centrifuge, pellets were washed twice by washing buffer I (3 mM EDTA, 0.2 mM EGTA, 1 mM DTT, 150 mM NaCl, protease inhibitor cocktail (Roche) and buffer II (3 mM EDTA, 0.2 mM EGTA, 1 mM DTT, 250 mM NaCl, protease inhibitor cocktail (Roche). After washing, pellets were sonicated and lysed by E1A lysis buffer. Then proteins were analyzed by Western's blot analysis.

### Immunofluorescence assay (IFA)

For IFA, cells were grown on chamber slides. After treated with indicated drugs, DU145 cells or PC-3 cells were then fixed with 4% paraformaldehyde and permeabilized in 0.2% Triton X-100 in PBS at room temperature. Cells were immunolabelled using anti-Skp2 antibodies (IFA: 1:100, Cell Signaling) or DAPI (4’,6-diamidino-2-phenylindole) and observed on Leica TCS SP5 confocal microscopy.

### Statistical analyses

All values were the means ± standard error of mean (SEM) of the replicate samples (n=3 to 6, depending on the experiment) and experiments were repeated by a minimum of three times. Differences between two groups were assessed using the unpaired two-tailed Student's *t*-test or by ANOVA if more than two groups were analyzed. The Tukey test was used as a post-hoc test in ANOVA for testing the significance of pairwise group comparisons. *P*-values <0.05 were considered statistically significant in all comparisons. SPSS version 13.0 for windows (LEAD technologies, Inc.) was used for all calculations.

## SUPPLEMENTARY MATERIALS FIGURES AND TABLES




