# Supplementary material for: Anti-cancer effect of danshen and dihydroisotanshinone I on prostate cancer: targeting the crosstalk between macrophages and cancer cells via inhibition of the STAT3/CCL2 signaling pathway
Source: Oncotarget. 2017 Feb 1;8(25):40246–63. doi: 10.18632/oncotarget.14958 (PMC5522253; doi:10.18632/oncotarget.14958)
Supplement: Supplementary file 1 [file oncotarget-08-40246-s001.pdf]

Anti-cancer effect of danshen and dihydroisotanshinone I on prostate cancer: targeting the crosstalk between macrophages and cancer cells via inhibition of the STAT3/CCL2 signaling pathway

SUPPLEMENTARY INFORMATION

MATERIALS AND METHODS

Cell culture and treatment

The human prostate cancer cell lines (DU145 cells, PC-3 cells and 22Rv1 cells) and mouse macrophage cell line (RAW264.7 cells) and human acute monocytic leukaemia cell line (THP-1 cells)) were obtained from the American Type Culture Collection. The DU145 cells, PC-3 cells and RAW264.7 cells were cultured in Dulbecco's Modified Eagle's medium (DMEM) (Invitrogen Corp., Carlsbad, CA), supplemented with 10% FBS at 37°C and 5% CO_2. The 22Rv1 cells and THP-1 cells were cultured in RPMI-1640 medium (RPMI) (Invitrogen Corp., Carlsbad, CA), supplemented with 10% fetal bovine serum (FBS) at 37°C and 5% CO_2. Dihydroisotanshinone I (DT) was obtained from ChemFaces Natural Products Co., Ltd., China (Catalog number: CFN-90162, the purity is 98% and its solubility in DMSO is >5mg/mL). Tanshinone I (TI) was obtained from was obtained from Sigma-Aldrich (Catalog number: SI-T5330). Tanshinone IIA (T2A) was obtained from was obtained from Sigma-Aldrich (Catalog number: SI-T4952). Salvianolic acid B (SA) was obtained from was obtained from Santa Cruz (Catalog number: sc-212911). MDV 3100 was obtained from Toronto Research Chemicals (Catalog number: M199800). Bicalutamide was obtained from Toronto Research Chemicals (Catalog number: B382000). DHT (dihydrotestosterone) was obtained from Sigma. Human prostate cancer cells and macrophages were cultured to 60-70% confluence prior to treatment. Medium was then replaced with fresh medium containing DT in DMSO (dimethyl sulfoxide) at the indicated concentrations. Cells treated with DMSO alone were used as untreated controls.

Invasion assay

Cell migration assays were performed as described previously [1]. Cell invasion was measured using Matrigel-coated film inserts (pore size, 8-µm) fitted into 24-well invasion chambers. DU145 cells (3×10^4 cells) were suspended in DMEM and added to the upper compartment of an invasion chamber in the presence or absence of DT or DMSO. DMEM with FBS was added to the lower chamber. The chambers were incubated at 37 °C in 5 % CO2. After 24 h, the filter inserts were removed, and the cells on the upper surfaces of the filters were removed with cotton swabs. The invasion of human prostate cancer cells was stained with crystal violet and was observed using Nikon TE3000 microscope. The numbers were averaged after counting 6 randomly selected fields. Each sample was assayed in triplicate, and each experiment was repeated at least twice.

Apoptosis assay

Human prostate cancer cells were seeded in a 100-mm plate and cultured overnight before treatment. Then, the cells were treated with control or 10 µM of DT for 24 hours. Then treated cells were detected by Annexin V-FITC Apoptosis Detection Kit (Strong Biotech Corporation, Cat No.AVK250) according to the manufacturer's instructions. In brief, at the end of the incubation period, the medium was removed. The treated cells were collected after washing by cold PBS. The supernatant was removed by centrifugation and then resuspended in FITC Annexin V binding buffer and PI by staining at room temperature in the dark for 15 min. The stained cells were analyzed by the flow cytometer BD FACSCanto (Becton Dickinson). Green fluorescence (530/42 nm), indicative of the annexin-VFITC binding of apoptotic cells, and red fluorescence (585/42 nm), indicative of PI uptake by damaged cells, were evaluated using logarithmic amplification and electronic compensation for spectral overlap. The amount of early apoptosis, late apoptosis, and necrosis were measured as the percentage of annexin-V positive/PI negative, annexin-V positive/PI positive, and annexin-V negative/PI positive cells, respectively. Each sample was assayed in triplicate, and each experiment was repeated at least twice.

Total RNA extraction and gene chip hybridization

Total RNA was extracted from all cell lines using RNeasy Mini kit (Qiagen, Valencia, CA, USA) and further purified with RNeasy Min-elute Clean-up Columns (Qiagen, Valencia, CA), as described by the manufacturers. RNA quantity and purity was assessed using NanoDrop ND-1000. Pass criteria for absorbance ratios was established as A260/A280 ≥ 1.8 and A260/A230 ≥ 1.5 indicating acceptable RNA purity. RIN values were ascertained using Agilent RNA 6000 Nano assay to determine RNA integrity. Pass criteria for RIN value was established at ≥ 6 indicating acceptable RNA integrity. Fluorescent aRNA targets were prepared from 1 or 2.5 μg total RNA samples using OneArray® Amino Allyl aRNA Amplification Kit (Phalanx Biotech Group, Taiwan) and Cy5 dyes (Amersham Pharmacia, Piscataway, NJ, USA). Fluorescent targets were hybridized to the Human Whole Genome OneArray® (HOA6.1) with Phalanx hybridization buffer using Phalanx Hybridization System. This array contains 30,275 DNA oligonucleotide probes, and each probe was a 60-mer designed in the sense direction. Among the probes, 29,187 probes correspond to the annotated genes in RefSeq v38 and Ensembl v56 database. Besides, 1,088 control probes were also included. After 16 hours' hybridization at 50°C, non-specific binding targets were washed away by three different washing steps (Wash I 42°C 5 mins; Wash II 42°C, 5 mins, 25°C 5 mins; Wash III rinse 20 times), and the slides were dried by centrifugation and scanned by Axon 4000B scanner (Molecular Devices, Sunnyvale, CA, USA). The intensities of each probe were obtained by GenePix 4.1 software (Molecular Devices).

Cytokine membrane array

The secretion medium (cell culture medium) of DU 145 cell lines were collected 24 hours after treating with indicated drugs. The secretion profiles of cytokines were measured using Human Cytokines array Panel A Array (R&D Systems, catalog number: ARY005), according to the manufacturer's instructions. Cell culture supernatants were mixed with a cocktail of biotinylated detection antibodies. Nitrocellulose membranes (spotted with different cytokine antibodies) were then incubated the sample/antibody mixture. After several washes, streptavidin-HRP and chemiluminescent detection reagents were added, which produced light at each spot proportional to the amount of cytokine bound.

Quantitative real time PCR

Total RNA was extracted from prostate cancer cells using the TRIzol reagent (Invitrogen, Cat. No. 15596-026) according to the manufacturer's instructions. Reverse transcription was performed using the Superscript first strand synthesis kit (Invitrogen, Number: 11904018). Quantitative real-time PCR analyses using the comparative CT method were performed on an ABI PRISM 7700 Sequence Detector System using the SYBR Green PCR Master Mix kit (Perkin Elmer, Applied Biosystems, Wellesley, MA, USA) according to the manufacturer's instructions. Following initial incubation at 50°C for 2 minutes and 10 minutes at 95°C, amplification was performed for 40 cycles at 95°C for 20 seconds, 65°C for 20 seconds and 72°C for 30 seconds. Primers used were: CCL2 forward, 5'-GTC TCT GCC GCC CTT CTG TG-3' and CCL2 reverse, 5'-GAC ACT TGC TGC TGG TGA TTC TTC-3'. Skp2 forward, 5'-TTA GTC GGG AGA ACT TTC CAG GTG-3' and skp2 reverse, 5'-AGT CAC GTC TGG GTG CAG ATTT-3'. RhoA forward, 5'-GAG CAC ACA AGG CGG GAG-3' and RhoA reverse, 5'-CTT GCA GAG CAG CTC TCG TAG-3'. SNAI1 forward, 5'-GAG GCG GTG GCA GAC TAG AGT-3' and SNAI1 reverse, 5'-CGG GCC CCC AGA ATA GTTC-3'. GAPDH forward, 5'-TGC ACC ACC AAC TGC TTAGC-3' and GAPDH reverse, 5'-GGC ATG GAC TGT GGT CATGA-3'. GAPDH was used as the housekeeping gene for data normalization.

Western blot analysis

Western blot analyses were performed as described previously [2]. For Western blotting, cellular extracts of the human prostate cancer cell line (DU145 cells and PC-3 cells) treated with DMSO or indicated concentrations of DT for 24 hours were prepared according to the manufacturer's instructions. Equal amounts of protein were fractionated on a 6 or 12% SDS-PAGE and transferred to polyvinylidene difluoride (PVDF) membranes. The membranes were then blocked with 5% nonfat dried milk for 30 minutes and incubated in primary antibody for 3 hours at room temperature. The primary antibodies used were: anti-phospho(Tyr705)-STAT3 antibody (p-STAT3)(Cell Signaling, ratio: 1:1000), anti-STAT3 antibody (Cell Signaling, ratio: 1:1000), anti-skp2 antibody (Cell Signaling, ratio: 1:1000), anti-β-actin antibody (Santa Cruz, IB: 1:10000) anti-α-tubulin antibody (Santa Cruz, IB: 1:10000) and anti-H3 antibody (Santa Cruz, IB: 1:10000). The primary antibodies and the secondary antibodies were diluted with 1% nonfat dried milk in 0.1% TBST (Tris-Buffered Saline Tween-20). Blots were washed by 0.1% TBST and incubated in horseradish peroxidase-conjugated secondary anti-mouse or anti-rabbit antibodies (Santa Cruz, ratio: 1:5000) for one hour at room temperature. After washing by 1X TBST again, protein signal was detected by chemiluminescence, using the Super Signal substrate (Pierce, Number: 34087).

REFERENCES

1. Liu F, Yu G, Wang G, Liu H, Wu X, Wang Q, Liu M, Liao K, Wu M, Cheng X and Hao H. An NQO1-initiated and p53-independent apoptotic pathway determines the anti-tumor effect of tanshinone IIA against non-small cell lung cancer. PloS one. 2012; 7:e42138.
2. Tsao CK, Cutting E, Martin J and Oh WK. The role of cabazitaxel in the treatment of metastatic castration-resistant prostate cancer. Therapeutic advances in urology. 2014; 6:97-104.

SUPPLEMENTARY FIGURES AND TABLE

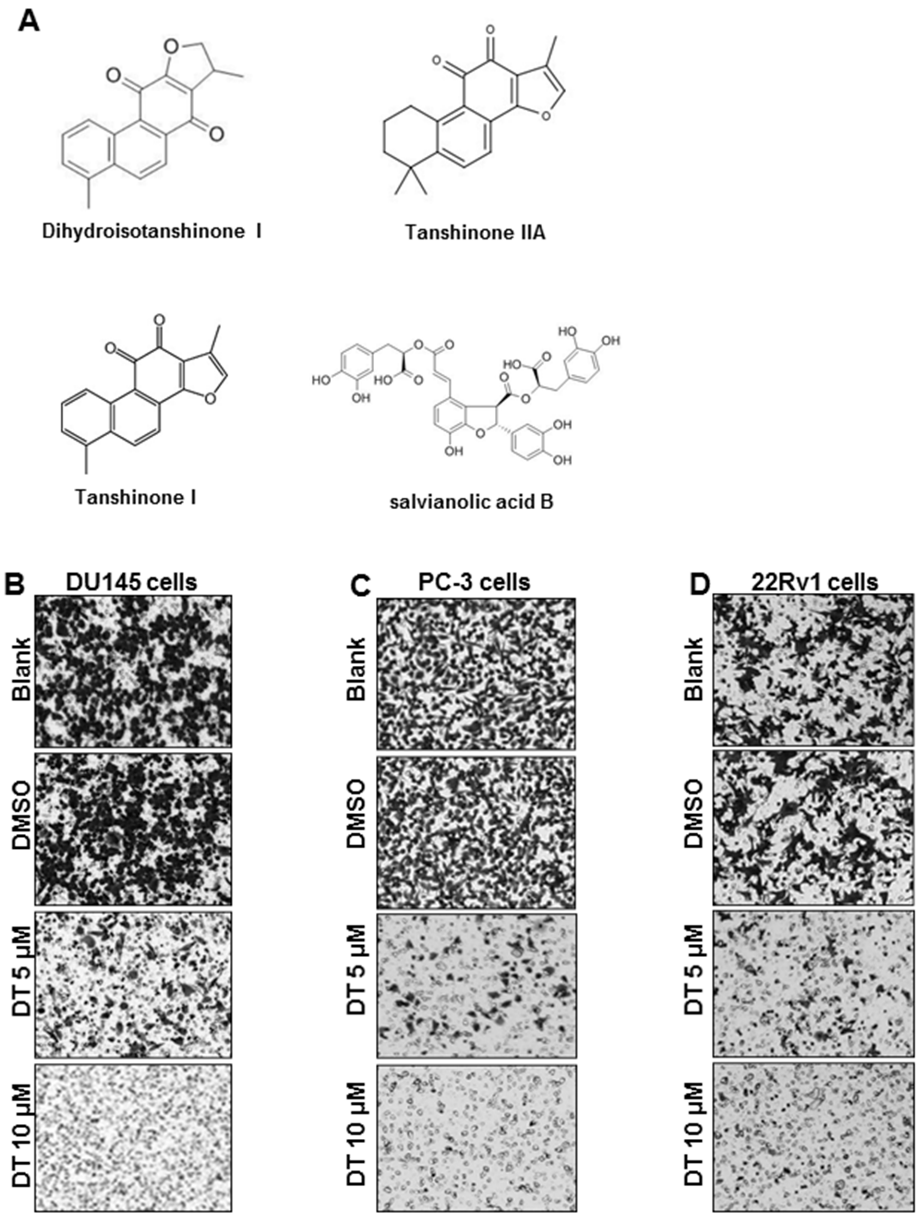

Supplementary Figure 1: DT block the human prostate cancer cells migration on *in vitro* wound healing assay and transwell migration assay and invasion assay. A. The structure of dihydroisotanshinone I, tanshinone IIA, tanshinone I and salvianolic acid B. The migration ability of DU145 cells **B.** PC-3 cells **C.** and 22Rv1 cells **D.** were measured by the transwell migration assay. After treated with indicated drugs for 24 hours, the photographs (× 100) were taken and the migratory cells were measured using AlphaEase®FC StandAlone Software. The mobility of DU145 cells. (*Continued*)

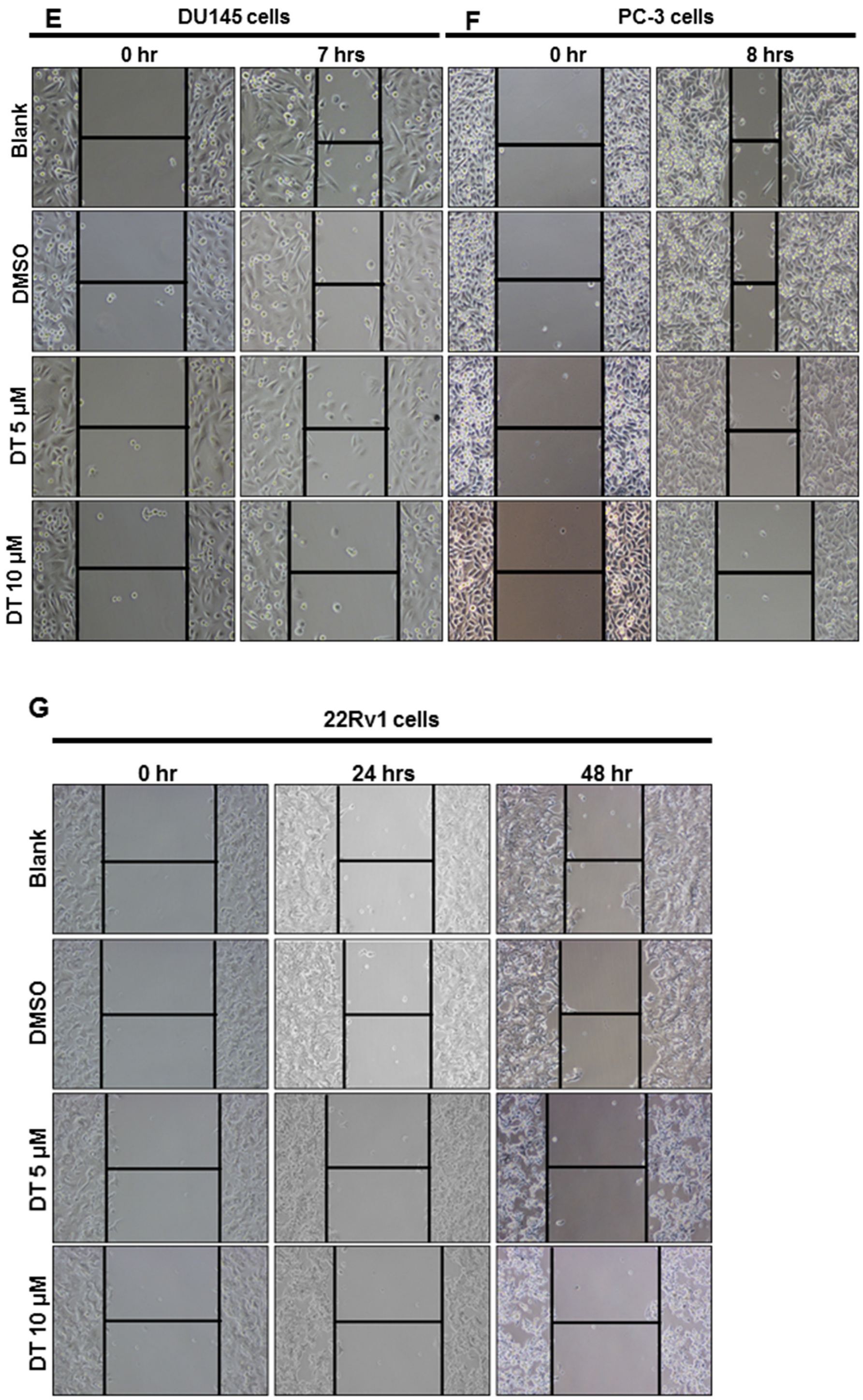

Supplementary Figure 1: (*Continued*) DT block the human prostate cancer cells migration on *in vitro* wound healing assay and transwell migration assay and invasion assay. E. PC-3 cells **F.** and 22Rv1 cells **G.** were measured by wound-healing assay. After treatment with indicated drugs, photographs (x100) were taken.

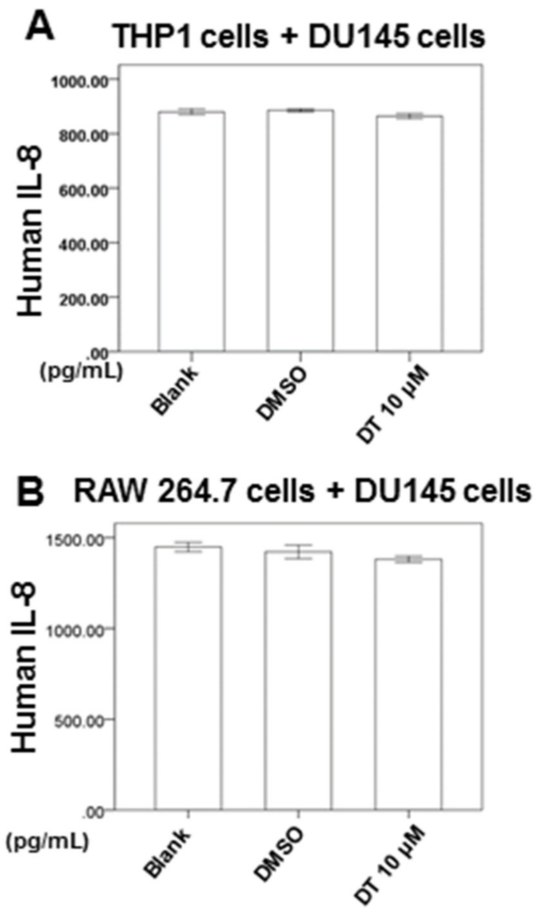

Supplementary Figure 2: Effects of DT on the IL-8 secretion from prostate cancer cells and macrophages co-culture *in vitro*. The conditioned medium of coculture with THP-1 cells/DU 145 cell **A.** or RAW 264.7 cells/DU 145 cells **B.** were collected from untreated cells and cells treated with DMSO or indicated drugs for 24 hours. The secretion of human IL-8 were measured by ELISA kits. All the results are representative of at least three independent experiments. (Error bars=mean±S.E.M).

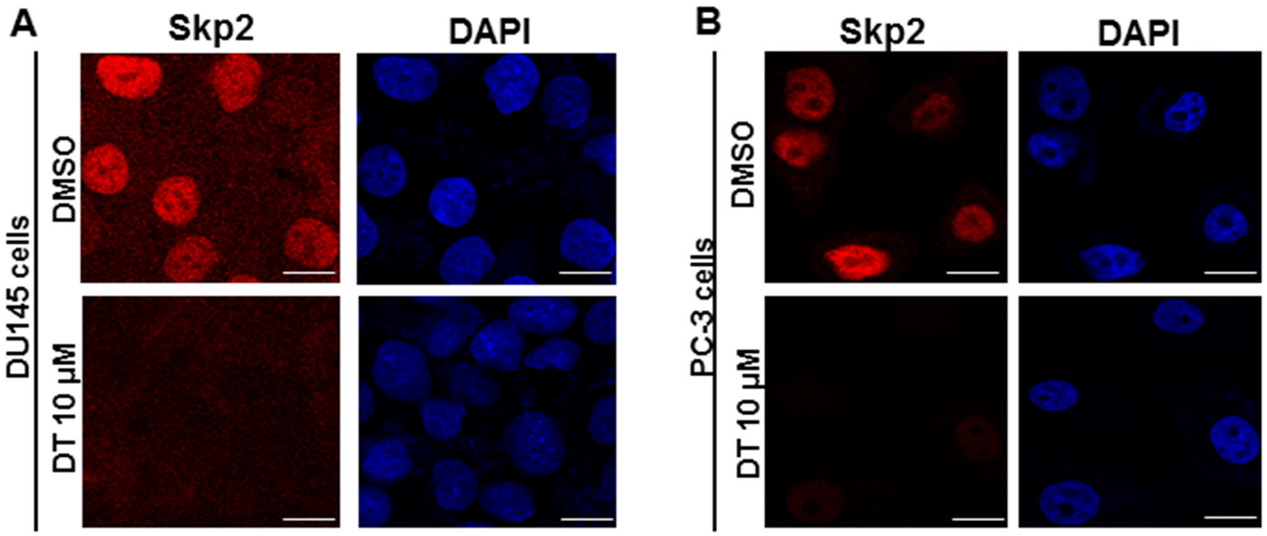

Supplementary Figure 3: DT inhibits the protein expression of Skp2. The expression of skp2 in DU145 cells **A.** or PC-3 cell **B.** after treatment with or without DT was detected by immunofluorescence staining with skp2 antibodies. Cells were stained with DAPI (right panel) and anti-skp2 antibody (left panel).

Supplementary Table 1: The gene ontology (GO) analysis based on biological process. The mRNA expression patterns of DU 145 cells under the treatment of DMSO or DT 10 µM for 24 hours were investigated by mRNA array. After gene ontology (GO) enrichment analysis based on biological process, the pathways and related genes were list.

See Supplementary File 1
